# Chemical characterization and antibacterial activities of Brazilian propolis extracts from *Apis mellifera* bees and stingless bees (*Meliponini*)

**DOI:** 10.1371/journal.pone.0307289

**Published:** 2024-07-16

**Authors:** Karolina Oliveira Gomes, Lorena Cristina Fernandes Messias da Silva, Rebeca Dias dos Santos, Bruno Alcântara Prado, Patrícia da Silva Montes, Letícia Fernandes Silva Rodrigues, Marta Oliveira de Araújo, Carla Azevedo Bilac, Daniel Oliveira Freire, Eliana Fortes Gris, Izabel Cristina Rodrigues da Silva, Lívia Cristina Lira de Sá Barreto, Daniela Castilho Orsi

**Affiliations:** 1 Laboratory of Quality Control and Post-Graduate Program in Health Sciences and Technologies, University of Brasília, Brasília, DF, Brazil; 2 Laboratory of Technologies and Post-Graduate Program in Health Sciences, University of Brasília, Brasília, DF, Brazil; 3 Laboratory of Quality Control, University of Brasília, Brasília, DF, Brazil; University of Messina, ITALY

## Abstract

The aim of this study was to evaluate the physicochemical composition and antibacterial activity of Brazilian propolis extracts from different types, concentrations, and extraction solvents and from different regions in Brazil. A total of 21 samples were analyzed, comprising 14 samples from *Apis mellifera* (12 green, 1 brown, and 1 red) and 7 samples from stingless bees (3 mandaçaia, 2 jataí, 1 hebora, and 1 tubuna). The analyses performed were dry extract, total phenolic content (TPC) and antioxidant activity (DPPH and ABTS). The antibacterial activity was performed by Determination of Minimal Inhibitory Concentration (MIC) and Minimal Bactericidal Concentration (MBC). The results showed that very low levels of phenolic compounds and antioxidant activity decreased the antimicrobial activity of the propolis extracts from tubuna and jataí. However, there was no correlation between the increase in propolis concentration in the extract, and the increase in antimicrobial activity. The highest TPC and antioxidant activity was obtained for green propolis extract made with 70% raw propolis that presented similar antibacterial activity to the samples formulated with 30% or less raw propolis. The aqueous propolis extract showed lower antimicrobial activity compared to the alcoholic extracts, indicating that ethanol is a better solvent for extracting the active compounds from propolis. It was observed that the MIC (0.06 to 0.2 mg/mL) and MBC (0.2 to 0.5 mg/mL) values for Gram-negative bacteria were higher compared to Gram-positive bacteria (MIC 0.001–0.2 mg/mL, and the MBC 0.02–0.5 mg/mL). The propolis extracts that exhibited the highest antimicrobial activities were from stingless bees hebora from the Distrito Federal (DF) and mandaçaia from Santa Catarina, showing comparable efficacy to samples 5, 6, and 7, which were the green propolis from the DF. Hence, these products can be considered an excellent source of bioactive compounds with the potential for utilization in both the pharmaceutical and food industries.

## Introduction

Currently, Brazil is the third largest global producer of propolis from *Apis mellifera* bees, second only to Russia and China. The annual production of Brazilian propolis is estimated to be 140–150 tons, with approximately 75% of this total being exported to Japan. Brazilian *Apis mellifera* bees are Africanized, exhibiting defensive characteristics and resistance to diseases, eliminating the need for chemical treatments seen in other countries. This makes Brazilian apicultural products of excellent quality and free from contamination [1–3].

Propolis is produced from resins collected from various plant parts (leaves, flowers, pollen, buds, and tree exudates), beeswax, pollen, and bee saliva. It presents itself as a malleable product used for sealing cracks and exhibiting antiseptic functions within the beehive [4]. Propolis has antimicrobial properties, including antibacterial, antifungal, and antiviral activities, as well as antioxidant activity [1, 5]

The biological activities of propolis are related to its chemical composition, which varies according to the flora visited by the bees. In addition to the propolis source, the extraction process associated with solvents modifies the properties of the propolis extract [1]. In Brazil, the most common process used in the preparation of propolis extract is the use of 70% ethanolic solution and maceration. The ratio of raw propolis to extract used is 1:3, meaning 1 part of propolis to 3 parts of ethanol, considering the production of a liquid extract with at least 11% (w/v) of dry matter of propolis [6].

The diverse types of Brazilian propolis exhibit a varied chemical composition according to their origin in different regions of the country. This variation is explained by the vast diversity of the Brazilian flora [4, 5]. In Brazil, the three most produced types of propolis are green propolis, red propolis, and brown propolis [1, 4, 5].

Most studies in the literature have investigated the biological activities of propolis produced by *Apis mellifera*. However, recently, several studies have explored the biological effects of propolis produced by other bees, such as stingless bees. The bee fauna of the *Meliponini* group (Hymenoptera: Apidae) is native to tropical and subtropical regions, distributed across more than 32 genera, with 244 species described in Brazil. This group of bees plays a crucial ecological role, contributing to the preservation of plant species through pollination [7].

In the last two decades, Meliponiculture has been gaining increasing visibility in the Brazilian scenario [8]. Stingless bees exhibit non-aggressive behavior, allowing colonies to be manipulated more easily compared to common honeybees [9]. Most stingless bee species are honey and propolis producers [10]. Promoting the management of native stingless bees contributes to forest conservation by maintaining the pollination process of plants. It also enables pollination of greenhouse crops and allows for beekeeping near residences, including urban areas. Meliponiculture is a sustainable and ecologically friendly activity, as bees are integral parts of the ecosystem. Moreover, it is economically viable, given that the honey produced by native bees is differentiated and has a guaranteed market [8].

Although many studies have explored the biological properties of Brazilian propolis, no research has been found that compares the antimicrobial and antioxidant activities among various types of Brazilian propolis extracts. These include green, red, and brown propolis from *Apis mellifera*, as well as propolis from mandaçaia, jataí, hebora, and tubuna stingless bees. The study also considers ethanolic and aqueous propolis extracts, extracts formulated with 70%, 30%, and less than 30% of raw propolis, and propolis from different regions of Brazil (the states of Distrito Federal and Goiás in the Midwest region, the state of Alagoas in the Northeast, the states of Paraná, Santa Catarina, and Rio Grande do Sul in the South, and the state of Minas Gerais in the Southeast). Therefore, the aim of this study was to evaluate the physicochemical composition and antibacterial activity of Brazilian propolis samples from different types, concentrations, and extraction solvents and from different regions in Brazil.

## Material and methods

### Samples of propolis *in natura* and propolis extracts

A total of 21 samples of extracts of propolis were analyzed, comprising 14 samples of propolis from *Apis mellifera* (12 green, 1 brown, and 1 red) and 7 samples of propolis from *Meliponini* stingless bees (3 from *Melipona quadrifasciata*, 2 from *Tetragonisca angustula*, 1 from *Tetragona clavipes*, and 1 from *Scaptotrigona bipunctata*). Six samples of propolis *in natura* were used, from which alcoholic extracts were prepared in the laboratory, and 15 samples of commercial propolis extracts were obtained from apiaries and “meliponarios” (stingless bee farms) in various regions of Brazil (Table 1). Raw propolis should be collected from beehives using stainless steel knives or spatulas and stored in clean, non-toxic plastic containers, labeled with the date and location of collection. The product is then taken for cleaning to remove foreign materials (dead bees, plant fragments, wax residues). After cleaning, the raw propolis should be placed in plastic containers and stored under refrigeration until processing or commercialization [6].

**Table 1:** Identification of extracts propolis samples.

Sample	Propolis	State and region of Brazil	Type	Extract description
Propolis extracts from *Apis mellifera* bees				
1	*in natura*	Goiás GO (Midwest)	green	Made in the laboratory
2	commercial	Goiás GO (Midwest)	green	Alcoholic extract *
3	*in natura*	Paraná PR (South)	green	Made in the laboratory
4	*in natura*	Distrito Federal DF (Midwest)	green	Made in the laboratory
5	commercial	Distrito Federal DF (Midwest)	green	Alcoholic extract *
6	commercial	Distrito Federal DF (Midwest)	green	Alcoholic extract *
7	commercial	Distrito Federal DF (Midwest)	green	Alcoholic extract *
8	commercial	Distrito Federal DF (Midwest)	green	Alcoholic extract **
9	*in natura*	Minas Gerais MG (Southeast)	green	Made in the laboratory
10	commercial	Minas Gerais MG (Southeast)	green	Alcoholic extract *
11	commercial	Minas Gerais MG (Southeast)	green	Alcoholic extract ***
12	commercial	Minas Gerais MG (Southeast)	green	Aqueous extract**
13	commercial	Santa Catarina SC (South)	brown	Alcoholic extract *
14	commercial	Alagoas AL (Nordeste)	red	Alcoholic extract *
**Propolis extracts from *Meliponini* stingless bee**				
15	*in natura*	Santa Catarina SC (South)	mandaçaia	Made in the laboratory
16	commercial	Rio Grande do Sul RS (South)	mandaçaia	Alcoholic extract *
17	commercial	Distrito Federal DF (Midwest)	mandaçaia	Alcoholic extract **
18	*in natura*	Paraná PR (South)	jataí	Made in the laboratory
19	commercial	Distrito Federal DF (Midwest)	jataí	Alcoholic extract **
20	commercial	Distrito Federal DF (Midwest)	heborá	Alcoholic extract *
21	commercial	Rio Grande do Sul RS (South)	tubuna	Alcoholic extract *

*Alcoholic extract, with a minimum of 11% (w/v) of dry extract as declared on the label

** Did not declare the percentage of dry extract on the label

*** Alcoholic extract, with a minimum of 24% (w/v) of dry extract as declared on the label. Mandaçaia (popular name of the bee) and *Melipona quadrifasciata* (scientific name of the bee); jataí (popular name of the bee) and *Tetragonisca angustula* (scientific name of the bee); hebora (popular name of the bee) and *Tetragona clavipes* (scientific name of the bee); tubuna (popular name of the bee) and *Scaptotrigona bipunctata* (scientific name of the bee).

To prepare the alcoholic extracts of propolis in the laboratory, 30 g of each sample of propolis *in natura* were macerated in a porcelain mortar with the aid of a pestle and the addition of 100 mL of ethanol 95%. After maceration, the material was kept under mechanical agitation for 18 hours, and then the solution was filtered through a cellulose filter [8].

### Physicochemical characterization of propolis extracts

The dry extract content was determined by drying the propolis extracts in a drying oven at 105°C until constant weight was obtained [11]. The total phenolic content (TPC) was determined using the Folin Ciocalteau method [12]. The quantity of total phenolic was expressed as Gallic acid equivalent (GAE) (g GAE/ 100 mL or %). The evaluation of the antioxidant activity was performed through the evaluation of free radicals capture DPPH (2,2-diphenyl-1-picrylhydrazyl) [13] and ABTS (2,2-azinobis-3-ethylbenzthiazoline-6-sulphonic acid) [14]. The results of antioxidant activity were expressed as Trolox equivalent (TE) (mM TE/mL). The tests were performed in triplicate and the results were expressed as mean and standard deviation.

### Bacterial strains used in antimicrobial activity tests and inoculum preparation

Antibacterial activity was tested using three Gram-positive strains: *Bacillus cereus* (ATCC 14579), *Staphylococcus aureus* (ATCC 25923), and *Streptococcus mutans* (ATCC 25175); and four Gram-negative strains: *Pseudomonas aeruginosa* (ATCC 27853), *Klebsiella pneumoniae* (ATCC BAA-1706), *Salmonella enterica* (ATCC 14028), and *Escherichia coli* (ATCC 25922). The bacterial inoculum was prepared by directly suspending microbial growth in Mueller Hinton broth with a turbidity equivalent to 0.5 on the McFarland scale (10^7^–10^8^ CFU/mL), adjusted to an optical density of 0.08–0.10 at 620 nm using a spectrophotometer [15]. All species of bacteria used in the present study can cause bacterial infections in humans, and some of these bacteria (notably *S*. *aureus*, *P*. *aeruginosa*, *K*. *pneumoniae* and *E*. *coli*) have currently developed significant antimicrobial resistance.

### Disk-diffusion assay of propolis extracts

Disk diffusion assay was performed by adding 10 μL of propolis extracts to 6 mm paper disks (Laborclin, Brazil). Petri dishes containing Mueller-Hinton agar were inoculated with 100 μL of bacterial inoculum using a sterile swab until a uniform smear was obtained. Disks impregnated with propolis extracts were applied to the plates containing the bacterial inoculum, and the Petri dishes were incubated for 24 hours at 37°C. Results were obtained by measuring the diameter of the growth inhibition zone and expressed in millimeters (mm). The tests were conducted in triplicate, and the results were presented as the mean and standard deviation [15].

### Determination of Minimal Inhibitory Concentration (MIC) and Minimal Bactericidal Concentration (MBC) of propolis extracts

The bacterial inoculum was diluted in Mueller-Hinton broth (1:150), resulting in a concentration of 10^6^ CFU/mL. Propolis extracts were diluted in Mueller-Hinton broth at concentrations of 0.05–0.20 mg/mL (MIC of Gram-negative strains), 0.001–0.20 mg/mL (MIC of Gram-positive strains), 0.15–0.50 mg/mL (MBC of Gram-negative strains), and 0.04–0.50 mg/mL (MBC of Gram-positive strains). Then, 0.1 mL aliquots of bacterial inoculum and 0.1 mL of propolis extracts, in triplicate, were pipetted into a 96-well microtiter plate. In the controls, for visualizing microbial growth, 0.1 mL of bacterial inoculum and 0.1 mL of Mueller-Hinton broth were pipetted into the microplate, and for visualizing the absence of microbial growth, 0.1 mL of undiluted propolis extracts and 0.1 mL of Mueller-Hinton broth were pipetted into the microplate. The microplates were incubated for 24 hours at 37°C. After incubation, to determine MBC, 0.1 ml of aliquots were pipetted onto Mueller-Hinton agar plates and spread using sterile Drigalsky loops. The plates were then incubated for 24 hours at 37°C. The MBC was determined at the lowest concentration of propolis extracts where no bacterial colonies were observed on the plates [16]. For the determination of MIC, it was necessary to adapt the methodology because the propolis extracts in aqueous solution (Mueller-Hinton broth) appeared milky and, consequently, exhibited high turbidity. Therefore, the conventional turbidity reading method [16] was unfeasible in tests involving propolis extracts. So, the methodology for determining MIC was modified according to Ristivojević et al. [17]. For MIC determination, after the incubation period, the colorimetric method using 0.01% (w/v) sodium resazurin ethanolic solution was employed. Ten microliters of the resazurin solution were applied to 0.1 mL of the aliquots for visual result interpretation, where the blue color indicated bacterial inactivity and the pink color indicated bacterial growth. Therefore, MIC was defined as the lowest concentration of propolis extracts inhibiting microbial growth and displaying a blue color in the presence of resazurin. Resazurin is a redox indicator used in cellular viability assessment. This reagent initially appears blue and becomes pink and fluorescent when reduced to resorufin by oxidoreductase enzymes present in live cells [18].

### Statistics

The results were expressed as the mean of triplicate analyses. The calculated p-value was obtained through the unpaired ANOVA test, and when the means were significantly different at p < 0.05, the Tukey test was used for mean comparisons. The data were analyzed using STATISTICA^®^ software, version 10.0.

## Results and discussion

### Physicochemical characterization of propolis extracts

Table 2 presents the results of the physicochemical characterization of propolis extracts. The Brazilian legislation [6] determines that ethanolic propolis extract must have a minimum of 11% (w/v) of dry extract, indicating that the extract was prepared with at least 30% (w/v) of propolis *in natura*. Most samples (n = 16, 76.19%) showed dry extract values above 11%, while 5 samples (23.81%) presented lower dry extract values of 5.56–7.99%. The sample that declared a minimum content of 24% dry extract on the label exhibited value of 24.03%, indicating that the extract was prepared with 70% (w/v) of propolis *in natura*. The aqueous propolis extract presented 38.84% of dry extract, and there is no minimum or maximum value established by Brazilian legislation for this type of propolis extract.

**Table 2:** Physicochemical characterization of propolis extracts.

Samples	Propolis extracts description	Analyses			
		Dry extract (%)	TPC (%)	DPPH (mM TE /mg)	ABTS (mM TE /mg)
	Propolis extracts from *Apis mellifera* bees				
1	Green *in natura* GO	13.60±0.35 a	3.17±0.14 a	119.96±2.33 a	243.42±13.95 a
2	Green commercial GO	13.36±0.00 a	1.54±0.02 b	94.11±1.75 b	275.91±7.45 b
3	Green *in natura* PR	13.69±0.01 a	2.42±0.08 c	106.03±4.30 c	239.04±30.37 a
4	Green *in natura* DF	11.70±0.53 b	1.79±0.28 d	108.70±4.09 c	200.89±24.03 c
5	Green commercial DF	14.14±1.18 c	2.89±0.81 e	101.63±1.42 c	256.80±14.14 d
6	Green commercial DF	12.71±0.04 b	2.06±0.16 f	92.75±2.04 b	184.50±33.83 e
7	Green commercial DF	7.99±0.56 d	2.13±0.09 f	84.05±0.16 d	161.60±27.47 f
8	Green commercial DF	5.84±2.17 d	1.76±0.07 d	80.48±0.45 d	151.19±25.44 f
9	Green *in natura* MG	12.45±0.07 b	2.86±0.01 e	115.07±6.19 e	287.14±26.66 g
10	Green commercial MG	12.22±0.04 b	1.85±0.07 d	116.65±2.88 e	295.21±12.88 g
11	Green commercial MG 70%	24.03±0.05 e	4.48±0.10 g	349.70±16.70 f	449.46±30.34 h
12	Green commercial MG aqueous	38.84±0.14 f	1.31±0.02 b	40.49±0.60 g	189.73±13.52 e
13	Brown commercial RS	12.61±0.21 b	1.86±0.07 d	111.92±2.56 a	274.78±21.42 b
14	Red commercial AL	7.90±0.10 d	0.86±0.02 h	53.96±1.00 g	186.83±22.76 e
	**Propolis extracts from *Meliponini* stingless bee**				
15	Mandaçaia *in natura* SC	12.09±2.06 b	0.73±0.07 h	22.25±0.02 h	42.33±7.45 i
16	Mandaçaia commercial RS	12.54±0.58 b	2.56±0.13 c	69.17±2,97 i	127.21±29.67 j
17	Mandaçaia commercial DF	5.56±0.48 d	0.88±0.02 h	17.14±0.38 h	35.32±4.54 i
18	Jataí *in natura* PR	12.43±0.19 b	0.12±0.13 i	0.20±0.04 j	2.22±0.14 l
19	Jataí commercial DF	5.61±0.43 d	0.10±0.15 i	0.12±0,03 j	1.88±0.39 l
20	Hebora commercial DF	12.24±0.27 b	2.66±0.01 c	162.53±16.41 l	424.02±12.73 h
21	Tubuna commercial RS	12.85±0.14 b	0.11±0.05 i	0.47±0.12 j	0.54±0.03 m

TPC = total phenolic content. Data are represented as mean ± SD. Different letters in the same row indicate significant differences (p < 0.05)

Contieri et al. [19] determined the dry extract content in commercial samples of green and brown propolis from the Southeast region of Brazil and obtained similar results, where 8 samples of ethanolic extracts presented 11.85–24.21% of dry extract and 4 samples of ethanolic extracts presented 4.79–10.60% of dry extract. The authors suggested that there is no efficient quality control for propolis extracts commercially available in Brazil. Furthermore, in the results obtained by Contieri et al. [19], the aqueous extract of green propolis presented 38.46% of dry extract. Nascimento et al. [20] evaluated 4 commercial extracts of brown and red propolis produced in the Northeast region of Brazil and obtained dry extract levels of 4.32%, 6.22%, 12.57% for brown propolis extracts and 13.69% for red propolis extract.

The recent update of Brazilian legislation [6] represented a step forward for stingless beekeepers in terms of regulating the production of bee products, including propolis. However, this legislation, which also encompasses bee products from *Apis mellifera*, established detailed hygienic, sanitary, and technological standards for apicultural establishments, but provided limited details on the quality criteria for propolis extracts. The legislation merely stated that these extracts must contain a minimum of 11% dry extract and that extracts produced with alcohol concentrations exceeding 70% should not be intended for consumption but may be used for other purposes.

The older regulatory directive [21] outlines the quality criteria for *Apis mellifera* propolis and its extracts, establishing minimum values for total phenolic content and antioxidant activity of these products. Therefore, in Brazil, there is still no official regulation for the minimum values of total phenolic compounds and antioxidant activity in propolis extracts from stingless bees. Ethanolic extracts of *Apis mellifera* propolis must contain a minimum of 0.50% of phenolic compounds [21]. All *Apis mellifera* propolis extracts presented total phenolic compounds values above 0.50%.

The sample prepared with 70% green propolis *in natura* (sample 11) showed the highest TPC value of 4.48%, while other samples containing green propolis exhibited TPC values ranging from 1.31% to 3.17% (samples 1–10 and 12). Green propolis stands out for its high TPC content, and this composition is attributed to its primary botanical origin. The primary botanical origin of this propolis is the apical buds and young leaves of *Baccharis dracunculifolia* DC. (Asteraceae), popularly known as “vassourinha do campo,” a native species widely spread in the Cerrado biome. Commercially in Brazil, green propolis is the most important, being mainly produced in the states of Minas Gerais and São Paulo (Southeast region of Brazil) [1, 22].

Similar results for TPC values of ethanolic extracts of Brazilian green propolis have been observed in the literature. Vieira et al. [23] observed a TPC value of 2.74% for green propolis extract collected in the state of Minas Gerais (Southeast region). Contieri et al. [19] obtained TPC values ranging from 0.81 to 1.89% for commercial samples of green propolis extracts. Salgueiro and Castro [24] obtained TPC value of 1.22% for commercial extract of green propolis prepared with 30% of raw propolis.

The lowest TPC value of propolis extracts from *Apis mellifera* bees was obtained for the red propolis extract (0.86%) (sample 14), and this probably occurred due to its low dry matter content (7.90%), as other studies in the literature have reported higher values for the TPC of Brazilian red propolis extracts (1.98–3.00%) [25, 26]. Red propolis is another type of propolis that has been attracting international market attention, produced by bees through the collection of resins from *Dalbergia ecastaphyllum* (L.) Taub. (Fabaceae). This propolis is found in the Northeast region of Brazil, especially in hives near mangroves in the states of Sergipe, Bahia, Alagoas, Paraíba, and Pernambuco [27, 28].

The brown propolis (sample 13) extract had a TPC value of 1.86%. Vieira et al. [23] obtained a content of total phenolic compounds for brown propolis extract of 1.19%. Tiveron et al. [29] observed that propolis extract from South region showed TPC value of 1.76%. Brazilian brown propolis is another commercially available type and is collected mainly in the South region of Brazil, which has native forests of *Araucaria angustifolia* (Bertol.) Kuntze (Araucariaceae). Thus, its botanical source appears to be mainly Araucaria, although some compounds found in it are also present in *B*. *dracunculifolia* [1, 3].

In relation to propolis extracts from stingless bees, there was a considerable variation in the values of total phenolic compounds. The extracts of propolis hebora (sample 20) and mandaçaia RS (sample 16) presented TPC values (2.56–2.66%) similar to some green propolis extracts. The samples 15 (mandaçaia *in natura* SC) and 17 (mandaçaia commercial DF) had a phenolic compound content of 0.73% and 0.88%. And the extracts of propolis from jataí (samples 18 and 19) and tubuna (sample 21) presented low quantification of phenolic compounds (0.10–0.12%). The native stingless bees *Melipona quadrifasciata*, *Tetragonisca angustula* and *Tetragona clavipes*, locally known as mandaçaia, jataí, and hebora, respectively, are widely distributed in Brazil [3, 30, 31]. The stingless bee species *Scaptotrigona bipunctata*, locally known as tubuna, is found in three Brazilian biomes (Pampa, Atlantic Forest, and Pantanal) [10].

Rocha et al. [32] reported that the two propolis extracts from the jataí bee species collected in the state of Bahia, northeastern Brazil, did not present TPC. Torres et al. [33] analyzed samples of stingless bee propolis obtained in the city of Rio das Antas, southern Brazil, and obtained TPC values of 0.38% for mandaçaia and 0.13% for jataí. Pazin et al. [34] reported a phenolic compound value of 1.25% for the propolis extract from hebora bees originating from the state of São Paulo. Piccinini et al. [10] observed a value of 0.95% phenolic compounds in the propolis extract from tubuna bees from the city of Gravataí –RS.

Regarding antioxidant activity, the highest values observed were for green propolis extracts made with 70% raw propolis (sample 11) (449.46 mM/mg for ABTS) and hebora (sample 20) (424.02 mM/mg for ABTS). The lowest antioxidant activity values were observed for jataí (samples 18 and 19) and tubuna (sample 21) (0.54–1.88 mM/mg for ABTS). Vieira et al. [23] observed an antioxidant value determined by ABTS of 293.90 mM/mg for the green propolis extract and of 109.29 mM/mg for the brown propolis extract, both collected in the state of Minas Gerais (Southeast region of Brazil). Machado et al. [26] reported that the best results for the antioxidant activity by DPPH was shown on the extract of green propolis from the state of Minas Gerais (Southeast region of Brazil) followed by the extract of red propolis from the state of Sergipe (Northeast region of Brazil). Torres et al. [33] reported that the mandaçaia propolis extract showed antioxidant activity 10 times superior in relation to the jataí propolis extract. Santos et al. [3] observed similar results in the DPPH free radical scavenging activity test, where the best result was observed with mandaçaia propolis extract, thus revealing its greater capacity to neutralize the action of free radicals than jataí propolis extracts.

The results of the present study support existing literature, where studies have shown a positive correlation between the quantity of phenolic compounds and the antioxidant activity of propolis extracts [24, 35]. Rocha et al. [32] observed a variety of phenolic compounds in the propolis extracts from mandaçaia bees (gallic acid 4.38 mg/L, caffeic acid 0.81 mg/L, p-coumaric acid 2.81 mg/L, ellagic acid 1.20 mg/L, rutin 1.52 mg/L, trans-cinnamic acid 1.60 mg/L, among others), while propolis extracts from jataí bees exhibited minimal phenolic compounds (epicatechin 0.15 mg/L, piceatannol 0.12 mg/L, resveratrol 0.06 mg/L, trans-cinnamic acid 0.08 mg/L).

### Disk-diffusion assay of propolis extracts

Propolis extracts were analyzed by the disk diffusion method with all bacteria tested in the study, however the Gram-negative bacteria did not show growth inhibition zones. Table 3 presents the results of the disk diffusion assay for Gram-positive bacterial strains. For *B*. *cereus*, sample 6 (commercial green propolis extract DF) and sample 20 (commercial hebora propolis extract DF) exhibited the largest inhibition zones (30.3–31.0 mm). For *S*. *aureus*, samples 5 and 6 (both commercial green propolis extracts DF) showed the largest zones of inhibition (46.0–49.0 mm), and for *S*. *mutans*, samples 4 and 5 (both green propolis extracts DF) presented the largest inhibition zones (45.0 mm).

**Table 3:** Disk diffusion assay for Gram-positive bacterial strains.

Samples	Propolis extracts description	*B*. *cereus* (mm)	*S*. *aureus* (mm)	*S*. *mutans* (mm)
Propolis extracts from *Apis mellifera* bees				
1	Green *in natura* GO	27.0±0.03 a	27.5±0.16 a	26.3±0.07 a
2	Green commercial GO	22.5±0.07 b	27.0±0.06 a	25.3±0.07 b
3	Green *in natura* PR	27.5±0.12 a	26.0±0.06 a	24.0±0.14 b
4	Green *in natura* DF	21.5±0.09 b	24.3±0.14 b	45.0±0.01 c
5	Green commercial DF	24.0±0.01 c	49.0±0.22 c	45.0±0.14 c
6	Green commercial DF	31.0±0.06 d	46.0±0.14 c	35.5±0.21 d
7	Green commercial DF	26.0±0.21 a	27.0±0.01 a	29.0±0.21 e
8	Green commercial DF	27.0±0.24 a	24.0±0.01 b	38.0±0.07 f
9	Green *in natura* MG	28.5±0.08 e	28.5±0.05 e	31.5±0.07 g
10	Green commercial MG	22.5±0.05 b	30.3±0.05 f	26.0±0.03 a
11	Green commercial MG 70%	23.3±0.08 b	29.6±0.18 f	26.0±0.01 a
12	Green commercial MG aqueous	29.0±0.03 e	30.0±0.08 f	21.8±0.05 h
13	Brown commercial RS	21.0±0.01 b	27.0±0.06 a	22.0±0.05 h
14	Red commercial AL	29.0±0.06 e	26.5±0.07 a	24.3±0.08 b
**Propolis extracts from *Meliponini* stingless bee**				
15	Mandaçaia *in natura* SC	23.0±0.01 b	29.0±0.01 f	24.0±0.14 b
16	Mandaçaia commercial RS	24.5±0.12 c	26.0±0.01 a	24.0±0.14 b
17	Mandaçaia commercial DF	24.0±0.14 c	28.0±0.11 e	30.0±0.14 g
18	Jataí *in natura* PR	n	n	n
19	Jataí commercial DF	n	n	n
20	Hebora commercial DF	30.3±0.07 d	26.0±0.01 a	24.0±0.14 b
21	Tubuna commercial RS	n	n	n

n = there was no zone of inhibition of microbial growth. Data are represented as mean ± SD. Different letters in the same row indicate significant differences (p < 0.05)

Nascimento et al. [20] evaluated 4 commercial extracts of brown and red propolis produced in the Northeast region of Brazil and obtained inhibition halos of 16.0–25.0 mm for *S*. *aureus* ATCC 25923. Silva et al. [36] reported that the ethanolic extracts of red propolis from the state of Alagoas (Northeast region of Brazil) showed inhibition halos of 10.7–15.7 mm for *S*. *aureus*. Custódio Reis et al. [37] reported that the ethanolic extracts of green propolis from the state of Minas Gerais (Southeast region of Brazil) showed inhibition halos of 16.6 mm for *Bacillus subtilis* ATCC 6051 and 22.0 mm for *S*. *aureus* ATCC 6538.

Abdullah et al. [38] determined the antimicrobial activities of propolis extracts produced by Brunei stingless bees *Geniotrigona thoracica*, *Heterotrigona itama*, and *Tetrigona binghami* and observed inhibition halos of 7.0–9.7 mm for *S*. *aureus* and 10.8–13.0 mm for *B*. *subtilis*. Okińczyc et al. [39] determined the antimicrobial activity of *Apis mellifera* and *Trigona* sp. propolis extracts from Nepal and observed inhibition halos of 24.0 mm for *S*. *aureus* ATCC 25923 and 19.0–20.0 mm for *B*. *subtilis*.

The extracts propolis of jataí (samples 18 and 19) and tubuna (sample 21) did not show inhibition halo for *B*. *cereus*, *S*. *aureus*, and *S*. *mutans*. This result may have occurred mainly due to the absence of phenolic compounds and low antioxidant activity in these propolis extracts, as these compounds are directly associated with the antibacterial activity of propolis [40]. Thus, the antimicrobial activity and chemical composition of propolis are directly linked to geographical location, biodiversity, bee species, and resin harvesting season [41].

The literature describes that propolis extracts exhibit higher antibacterial activity against Gram-positive bacteria compared to Gram-negative bacteria. This phenomenon is attributed to the difference in cell wall composition between Gram-negative and Gram-positive bacteria [40, 42]. Gram-negative bacteria have an outer membrane rich in lipopolysaccharides that restricts the diffusion of hydrophobic compounds through it, whereas Gram-positive bacteria lack this outer membrane, allowing hydrophobic constituents to infiltrate the cell membrane. This infiltration leads to increased ionic permeability, leakage of intracellular content, and eventual cell death. However, hydrophobic compounds can still exert antimicrobial activity against Gram-negative bacteria, as outer membrane porin proteins form channels with sufficient size to allow the passage of small molecular weight compounds [43].

Table 4 presents the results of determination of Minimal Inhibitory Concentration (MIC) and Minimal Bactericidal Concentration (MBC) of propolis extracts for Gram-positive bacterial strains. The propolis extracts that exhibited the lowest MIC and MBC values for *B*. *cereus*, *S*. *aureus*, and *S*. *mutans*, and therefore the highest antibacterial activities, were samples 5, 6, and 7 (green commercial DF), 9 (green *in natura* MG), 15 (mandaçaia *in natura* SC), and 20 (hebora commercial DF) (MIC from 0.001 to 0.005 mg/mL and MBC from 0.02 to 0.15 mg/mL). On the other hand, propolis extracts that demonstrated the lowest antibacterial activities for *B*. *cereus*, *S*. *aureus*, and *S*. *mutans* were samples 12 (green commercial MG aqueous) and 21 (tubuna commercial RS) (MIC from 0.06 to 0.2 mg/mL and MBC from 0.1 to 0.5 mg/mL).

**Table 4:** Minimal Inhibitory Concentration (MIC) and Minimal Bactericidal Concentration (MBC) of propolis extracts for Gram-positive bacterial strains.

Samples	Propolis extracts description	Gram-positive bacterial strains					
		*B*. *cereus*		*S*. *aureus*		*S*. *mutans*	
		MIC (mg/mL)	MBC (mg/mL)	MIC (mg/mL)	MBC (mg/mL)	MIC (mg/mL)	MBC (mg/mL)
	Propolis extracts from *Apis mellifera* bees						
1	Green *in natura* GO	0.002 a	0.040 a	0.001 a	0.200 a	0.002 a	0.200 a
2	Green commercial GO	0.025 b	0.100 b	0.025 b	0.340 b	0.053 b	0.200 a
3	Green *in natura* PR	0.001 a	0.040 a	0.003 a	0.200 a	0.004 a	0.150 b
4	Green *in natura* DF	0.004 a	0.030 a	0.003 a	0.150 c	0.004 a	0.150 b
5	Green commercial DF	0.001 a	0.040 a	0.001 a	0.080 d	0.003 a	0.080 c
6	Green commercial DF	0.005 a	0.020 a	0.004 a	0.080 d	0.005 a	0.080 c
7	Green commercial DF	0.001 a	0.040 a	0.001 a	0.080 d	0.003 a	0.080 c
8	Green commercial DF	0.005 a	0.030 a	0.004 a	0.100 d	0.010 c	0.100 c
9	Green *in natura* MG	0.002 a	0.020 a	0.004 a	0.100 d	0.003 a	0.100 c
10	Green commercial MG	0.025 b	0.150 c	0.017 c	0.340 b	0.053 b	0.150 b
11	Green commercial MG 70%	0.025 b	0.150 c	0.017 c	0.340 b	0.053 b	0.150 b
12	Green commercial MG aqueous	0.100 c	0.150 c	0.200 d	0.500 e	0.100 d	0.500 d
13	Brown commercial RS	0.025 b	0.150 c	0.025 b	0.340 b	0.053 b	0.200 a
14	Red commercial AL	0.025 b	0.080 b	0.025 b	0.200 a	0.033 e	0.200 a
**Propolis extracts from *Meliponini* stingless bee**							
15	Mandaçaia *in natura* SC	0.005 a	0.020 a	0.005 a	0.080 d	0.005 a	0.150 b
16	Mandaçaia commercial RS	0.005 a	0.020 a	0.020 c	0.100 d	0.010 c	0.100 c
17	Mandaçaia commercial DF	0.005 a	0.020 a	0.060 e	0.200 a	0.030 e	0.250 e
18	Jataí *in natura* PR	0.020 b	0.100 b	0.080 f	0.300 b	0.060 f	0.300 f
19	Jataí commercial DF	0.030 b	0.100 b	0.130 g	0.500 e	0.080 g	0.250 e
20	Hebora commercial DF	0.001 a	0.020 a	0.003 a	0.080 d	0.007 a	0.080 c
21	Tubuna commercial RS	0.060 c	0.100 b	0.200 d	0.500 e	0.200 h	0.500 d

Data are represented as mean ± SD. Different letters in the same row indicate significant differences (p < 0.05)

### Minimal Inhibitory Concentration (MIC) and Minimal Bactericidal Concentration (MBC) of propolis extracts

Table 5 presents the results of MIC and MBC of propolis extracts for Gram-negative bacterial strains. It was observed that the MIC (0.06 to 0.2 mg/mL) and MBC (0.2 to 0.5 mg/mL) values for Gram-negative bacteria were higher compared to Gram-positive bacteria (MIC values ranged from 0.001 to 0.2 mg/mL, and the MBC values ranged from 0.02 to 0.5 mg/mL). The sample 21 (tubuna commercial RS), which exhibited lower antimicrobial activity against Gram-positive bacteria, showed MIC (0.06 to 0.2 mg/mL) and MBC (0.2 to 0.5 mg/mL) values for Gram-negative bacteria comparable to other propolis extracts in the study. On the other hand, sample 12 (green commercial MG aqueous) did not demonstrate bactericidal activity against the tested Gram-negative bacteria.

**Table 5:** Minimal Inhibitory Concentration (MIC) and Minimal Bactericidal Concentration (MBC) of propolis extracts for Gram-negative bacterial strains.

Samples	Propolis extracts description	Gram-positive bacterial strains							
		*P*. *aeruginosa*		*K*. *pneumoniae*		*S*. *enterica*		*E*. *coli*	
		MIC	MBC	MIC	MBC	MIC	MBC	MIC	MBC
		(mg/mL)							
	Propolis extracts from *Apis mellifera* bees								
1	Green *in natura* GO	0.08 a	0.25 a,d	0.15 a	0.35 a	0.15 a	0.35 a	0.10 a	0.35 a
2	Green commercial GO	0.13 b	0.35 b	0.15 a	0.35 a	0.16 a	0.50 b	0.15 b	0.20 b
3	Green *in natura* PR	0.12 b	0.25 a,d	0.15 a	0.35 a	0.15 a	0.35 a	0.10 a	0.20 b
4	Green *in natura* DF	0.12 b	0.35 b	0.12 b	0.25 b,c	0.12 b	0.25 c,d	0.10 a	0.20 b
5	Green commercial DF	0.08 a	0.20 a	0.13 b	0.20 b	0.12 b	0.20 c	0.08 a	0.20 b
6	Green commercial DF	0.08 a	0.20 a	0.13 b	0.30 c	0.12 b	0.25 c,d	0.08 a	0.20 b
7	Green commercial DF	0.08 a	0.25 a,d	0.13 b	0.20 b	0.12 b	0.25 c,d	0.10 a	0.20 b
8	Green commercial DF	0.08 a	0.50 c	0.20 c	0.50 d	0.15 a	0.30 d	0.10 a	0.20 b
9	Green *in natura* MG	0.12 b	0.35 b	0.12 b	0.25 b,c	0.12 b	0.25 c,d	0.10 a	0.25 b
10	Green commercial MG	0.16 c	0.35 b	0.15 a	0.35 a	0.08 c	0.50 b	0.06 c	0.20 b
11	Green commercial MG 70%	0.15 c	0.25 a,d	0.15 a	0.25 b,c	0.16 a	0.50 b	0.05 c	0.35 a
12	Green commercial MG aqueous	>0.16	n	>0.16	n	>0.16	n	>0.16	n
13	Brown commercial RS	0.13 b	0.35 b	0.15 a	0.35 a	0.08 c	0.50 b	0.15 b	0.20 b
14	Red commercial AL	0.08 a	0.20 a	0.15 a	0.20 b	0.16 a	0.20 c	0.10 a	0.20 b
	**Propolis extracts from *Meliponini* stingless bee**								
15	Mandaçaia *in natura* SC	0.13 b	0.30 d	0.13 b	0.30 c	0.13 b	0.30 d	0.06 c	0.20 b
16	Mandaçaia commercial RS	0.13 b	0.20 a	0.10 d	0.30 c	0.10 c	0.30 d	0.08 a	0.20 b
17	Mandaçaia commercial DF	0.13 b	0.30 d	0.20 c	0.30 c	0.10 c	0.30 d	0.10 a	0.20 b
18	Jataí *in natura* PR	0.13 b	0.30 d	0.13 b	0.30 c	0.20 d	0.30 d	0.10 a	0.30 c
19	Jataí commercial DF	0.20 d	0.50 c	0.13 b	0.50 d	0.20 d	0.50 b	0.08 a	0.50 d
20	Hebora commercial DF	0.05 e	0.25 a,d	0.20 c	0.30 c	0.15 a	0.20 c	0.10 a	0.30 c
21	Tubuna commercial RS	0.10 a	0.30 d	0.20 c	0.30 c	0.15 a	0.50 b	0.15 b	0.30 c

n = there was no antibacterial activity. Data are represented as mean ± SD. Different letters in the same row indicate significant differences (p < 0.05)

The commercial aqueous extract of propolis (prepared with green propolis from the state of Minas Gerais in the Southeast region of Brazil) (sample 12) exhibited lower antimicrobial activity in CIM and CBM tests compared to other ethanolic propolis extracts. This result highlights that the solvent has a direct influence on the effectiveness of propolis as an antimicrobial agent. Ethyl alcohol is the most widely used solvent for the preparation of propolis extracts, due to the nonpolar properties of most substances that compose propolis. Thus, propolis, being derived from vegetable resins, has low solubility in water, but high solubility in organic solvents [44, 45]. Sun et al. [46] analyzed the effect of different concentrations of hydroalcoholic solutions (ethanol at 25, 50 and 75%) on the extraction of phenolic compounds and flavonoids from raw propolis. They observed that the higher the concentration of ethanol used, the greater the extraction of these compounds.

In the literature, different techniques for preparing the aqueous extract of propolis have been described. Kubiliene et al. [47] conducted maceration of ground propolis in water at room temperature and filtered to obtain the aqueous extract of propolis (W1). They also performed maceration of ground propolis in water heated to 70°C, with the addition of propylene glycol, and filtered to obtain the aqueous extract of propolis (W2). The propolis extract with water only (W1) showed no antimicrobial activity, while the propolis extract W2 demonstrated antimicrobial activity in the disk diffusion test against *S*. *aureus* (15.8 mm) and *B*. *cereus* (17.2 mm). Rocha et al. [48] described the preparation of the aqueous propolis extract by macerating ground propolis in a 70% hydroalcoholic solution, followed by filtration of the extract. The obtained extract was concentrated using a rotary evaporator at temperatures of 40-60°C. After solvent evaporation (80–90% dry matter), the soft propolis extract underwent alkaline hydrolysis and was then solubilized in water. The aqueous propolis extract exhibited bactericidal activity against the tested bacteria (*S*. *aureus* ATCC 25923, *S*. *epidermidis* ATCC 14990, *S*. *pneumoniae* ATCC 49619, and *E*. *coli* ATCC 25922).

In the results obtained for the ethanolic extract of green propolis prepared with 70% raw propolis (sample 11) (green propolis from the state of Minas Gerais in the Southeast region of Brazil), its high concentration of dry extract did not result in greater antibacterial activity compared to other ethanolic extracts of green propolis prepared with 30% raw propolis. This is an important finding, considering that the cost of the 70% raw propolis extract is twice that of the extracts with 30% raw propolis. One hypothesis for the equivalent antimicrobial activity between extracts with 30% and 70% raw propolis is that a high concentration of propolis makes the extract less soluble in an aqueous medium, causing greater difficulty in diffusion and action against microorganisms.

Thus, it became evident that very low levels of phenolic compounds and antioxidant activity impaired the antimicrobial activity of propolis extracts, as was the case for tubuna (sample 21) (in disk diffusion, MIC, and MBC tests) and jataí (samples 18 and 19) (in disk diffusion test) propolis extracts. On the other hand, there was no linear correlation between the increase in propolis concentration in the extract, which consequently increased the levels of TPC and antioxidant activity, and the increase in antimicrobial activity, as was the case for sample 11 (green commercial MG 70%). Sample 7 (green commercial DF), despite being formulated with less than 30% raw propolis, stood out for its good antimicrobial activity. Other samples in the study, also formulated with less than 30% raw propolis (8 green commercial DF, 14 red commercial AL, and 17 mandaçaia commercial DF), demonstrated antimicrobial activity comparable to other propolis extracts in the study. Thus, it was observed that propolis extracts with quantities below 30% raw propolis also exhibited good antimicrobial activity.

In the literature, many studies have produced propolis extracts below the concentration of 30% of raw propolis. Machado et al. [26] prepared extracts from red propolis from the state of Sergipe (Northeast region), green propolis from the state of Minas Gerais (Southeast region), and brown propolis from the state of Santa Catarina (South region) with 13.3% of raw propolis. They obtained MIC values of 0.05–0.4 mg/mL and MBC values of 0.8–1.0 mg/mL for *S*. *aureus* ATCC 25923, and for *E*. *coli* ATCC 25922, MIC values of 0.4–0.8 mg/mL and MBC values of 0.8->1.6 mg/mL. Tiveron et al. [29] prepared propolis extracts from the Southern region of Brazil with 8% of raw propolis and obtained the lowest MIC values (0.01–0.1 mg/mL) for Gram-positive bacteria (*S*. *mutans*, *S*. *oralis*, and *S*. *aureus*). The propolis extracts were also effective against *P*. *aeruginosa* (Gram-negative), with MIC values ranging from 0.1 to 0.2 mg/mL. Barreto et al. [25] prepared extracts from red propolis from the state of Alagoas (Northeast region) with 8% of raw propolis and found MIC values of 0.006 to 0.02 mg/mL for *S*. *aureus* and 0.02–0.09 mg/mL for *E*. *coli*. It is noteworthy that there was a higher antibacterial activity of Brazilian propolis extracts from *Apis mellifera* against Gram-positive bacteria compared to Gram-negative bacteria.

In relation to propolis extracts from stingless bees, Santos et al. [3] reported a MIC value of 0.25 mg/mL for *S*. *aureus* and a MIC value of 0.5 mg/mL for *P*. *aeruginosa* for the ethanolic extract of mandaçaia propolis from the city of Blumenau, Santa Catarina, South region of Brazil. The extract of jataí propolis was not tested due to the low content of phenolic compounds. Torres et al. [33] compared the ethanolic extracts of propolis from mandaçaia and jataí collected in the South region of Brazil and Gram-positive bacteria (*E*. *faecalis*, *S*. *aureus* and MRSA) were more sensitive than Gram-negative bacteria (*E*. *coli* and *K*. *pneumoniae*) to both propolis extracts. The mandaçaia propolis extract was more effective against the bacteria tested than the jataí propolis extract.

Isidorov et al. [49] analyzed the propolis extract from the hebora bee in Argentina and reported MIC values of 0.03 mg/mL and MBC of 0.13 mg/mL for *S*. *aureus*, MIC of 0.13 mg/mL and MBC of 0.50 mg/mL for *E*. *coli*, and MIC of 0.50 mg/mL and MBC >2.00 mg/mL for *P*. *aeruginosa*. Surek et al. [50] analyzed propolis extracts from tubuna and mandaçaia bees collected in the state of Paraná, Southern Brazil, reporting MIC value of 0.25 mg/mL for mandaçaia propolis extract and inefficacy of the tubuna propolis extract (MIC >1.0 mg/mL) against *S*. *aureus*. None of the propolis extracts were effective against Gram-negative bacteria (*E*. *coli*, *K*. *pneumoniae*, and *P*. *aeruginosa*), with MIC values >1.0 mg/mL. Thus, it was observed that stingless bees also exhibit greater antibacterial activity against Gram-positive bacteria compared to Gram-negative bacteria.

The literature indicates that the mechanism of action of propolis is not determined by the isolated action of its bioactive constituents, but rather by a complex interaction among its various chemical compounds that synergistically contribute to the antimicrobial activity of propolis [51]. According to Przybyłek and Karpinski [40], several mechanisms of action are proposed to explain the antimicrobial activity exerted by propolis, such as inhibition of cell division and synthesis of nucleic acids and proteins, enzyme inactivation, alteration of the permeability of the bacterial cytoplasmic membrane, and bacteriolysis.

In Brazil, the Federal District is still relatively unknown for propolis production, despite its entire territory being situated in the Cerrado biome where there is a high incidence of *Baccharis dracunculifolia* [52]. Thus, in this study, we observed that green propolis extracts from the Federal District (samples 5, 6, and 7) stood out for their high antimicrobial activity. The production of bee products is growing in the Federal District, with currently 80 beekeepers registered and producing 13.8 tons of honey in 2019, along with other derivatives such as propolis, wax, and pollen [53].

Another noteworthy result was that the propolis extracts from mandaçaia *in natura* SC (sample 15), and hebora commercial DF (sample 20) exhibited comparable efficacy to samples 5, 6, and 7, which were the green propolis extracts with the highest antimicrobial activity in the study. Studies on propolis from different species of stingless bees are still scarce in the literature when compared to propolis from *Apis mellifera*. Propolis from stingless bees has a varied chemical composition according to bee species and, especially, the plant source. Thus, stingless bees use different plants for propolis production, resulting in propolis with distinct chemical compositions [54].

According to Isidorov et al. [49], it is likely that stingless bees do not exhibit strong selectivity in resin collection and use resources provided by local vegetation for propolis production, resulting in significant differences in the chemical composition of hebora bee propolis in Argentina and Brazil. Studies by Surek et al. [50] and Torres et al. [33] on the mandaçaia bee propolis also did not show similarities in chemical composition, with some of the common chemical compounds detected by the authors being p-coumaric acid, gallic acid, and aromadendrin. Despite the different chemical compositions, propolis from stingless bees contains phenolic compounds on a larger scale, as well as terpenes and saponins that exhibit antimicrobial activity [54].

In conclusion, the results obtained in this study demonstrated high antimicrobial activity and antioxidant activity for several types of Brazilian propolis, and thus, there is the possibility of diverse applications of these products in both the food and pharmaceutical industries, as food preservatives, adjuvants in antimicrobial therapy against resistant bacteria, and antiseptic and sanitizing pharmaceutical formulations.
